# A Parent Coach–Led Model of Well-Child Care for Young Children in Low-Income Communities: Protocol for a Cluster Randomized Controlled Trial

**DOI:** 10.2196/27054

**Published:** 2021-11-25

**Authors:** Rachel Hurst, Kendra Liljenquist, Sarah J Lowry, Peter G Szilagyi, Kevin A Fiscella, Marcia R Weaver, Lorena Porras-Javier, Janette Ortiz, Laura J Sotelo Guerra, Tumaini R Coker

**Affiliations:** 1 School of Public Health New York University New York, NY United States; 2 Seattle Children's Research Institute Seattle, WA United States; 3 Department of Pediatrics University of Washington School of Medicine Seattle, WA United States; 4 Department of Pediatrics University of California, Los Angeles Los Angeles, CA United States; 5 Department of Family Medicine University of Rochester Medical Center Rochester, NY United States; 6 Department of Health Metrics Sciences School of Medicine University of Washington Seattle, WA United States; 7 Institute for Health Metrics and Evaluation School of Medicine University of Washington Seattle, WA United States

**Keywords:** preventive care, well-child care, community health centers

## Abstract

**Background:**

The Parent-focused Redesign for Encounters, Newborns to Toddlers (PARENT) intervention was created as a team-based approach to well-child care (WCC) that relies on a health educator (*Parent Coach*) to provide the bulk of WCC services, address specific needs faced by families in low-income communities, and decrease reliance on the clinician as the primary provider of WCC services.

**Objective:**

This study aims to evaluate the impact of PARENT using a cluster randomized controlled trial.

**Methods:**

This study tested the effectiveness of PARENT at 10 clinical sites in 2 federally qualified health centers in Tacoma, Washington, and Los Angeles, California. We conducted a cluster randomized controlled trial that included 916 families with children aged ≤12 months at the time of the baseline survey. Parents will be followed up at 6 and 12 months after enrollment. The Parent Coach, the main element of PARENT, provides anticipatory guidance, psychosocial screening and referral, developmental and behavioral surveillance, screening, and guidance at each WCC visit. The coach is supported by parent-focused previsit screening and visit prioritization, a brief, problem-focused clinician encounter for a physical examination and any concerns that require a clinician’s attention, and an automated text message parent reminder and education service for periodic, age-specific messages to reinforce key health-related information recommended by Bright Futures national guidelines. We will examine parent-reported quality of care (receipt of nationally recommended WCC services, family-centeredness of care, and parental experiences of care), and health care use (WCC, urgent care, emergency department, and hospitalizations), conduct a cost analysis, and conduct a separate time-motion study of clinician time allocation to assess efficiency. We will also collect data on exploratory measures of parent-and parenting-focused outcomes. Our primary outcomes were receipt of anticipatory guidance and emergency department use.

**Results:**

Participant recruitment began in March 2019. After recruitment, 6- and 12-month follow-up surveys will be completed. As of August 30, 2021, we enrolled a total of 916 participants.

**Conclusions:**

This large pragmatic trial of PARENT in partnership with federally qualified health centers will assess its utility as an evidence-based and financially sustainable model for the delivery of preventive care services to children in low-income communities.

**Trial Registration:**

ClinicalTrials.gov: NCT03797898; https://clinicaltrials.gov/ct2/show/NCT03797898

**International Registered Report Identifier (IRRID):**

DERR1-10.2196/27054

## Introduction

### Background

Well-child care (WCC) visits for child preventive care during the first 3 years of life are important opportunities to address social, developmental, behavioral, and health concerns of young children and their families [1]. Despite the great potential of WCC to positively impact child health and well-being, multiple studies have demonstrated that many children do not receive all recommended preventive and developmental services during these visits, and that most parents do not have all of their psychosocial, developmental, and behavioral concerns addressed at these WCC visits [2-8]. Unfortunately, this unmet need is often the greatest for low-income families [9] as they often have substantial needs.

In the United States, WCC is not optimally structured to meet the vast array of preventive care needs that families in low-income communities often have [8]. Major structural problems with WCC include (1) reliance solely on busy clinicians (pediatricians, family physicians, or nurse practitioners) for most basic, routine WCC services [10-13], (2) limited to brief (often 15- to 20-minute) face-to-face clinician-directed WCC visits to address the wide array of education and guidance services in WCC [1,7,14]; (3) the need for high-level clinicians to focus clinical time on patients with complex medical needs, and (4) lack of a systematic, patient-driven method for visit customization to best meet families’ needs [15,16]. These problems in the structure of WCC are a key contributor to the wide variations in both, the processes of WCC and the receipt of preventive care services. This can lead to suboptimal quality of WCC services, resulting in missed opportunities to intervene and support the health and well-being of children in low-income communities.

To address the gaps in current WCC, we partnered with federally qualified health centers (FQHCs) to develop a new model of WCC to meet the needs of children in low-income communities. The Parent-focused Redesign for Encounters, Newborns to Toddlers (PARENT) intervention is a team-based approach to WCC relying on a health educator (*Parent Coach*) to provide the bulk of WCC services, address specific needs faced by families in low-income communities, and decrease reliance on the clinician as the primary provider of WCC services [17-19].

Although several strategies to redesign the structure of WCC have been proposed and studied, there are few evidence-based comprehensive models that are financially sustainable alternatives to the current WCC [11,20]. In a systematic review of tools and strategies for WCC clinical practice redesign for young children in the United States, we highlighted 17 published articles that focused on interventions to improve WCC delivery [11]. In this review, and a more recent update of the review, we identified 2 WCC practice-based interventions (PARENT and Healthy Steps for Young Children) in which a nonclinician was added as part of the WCC team to enhance preventive care services for young children [8,11]. These 2 WCC practice interventions demonstrate the effectiveness of using a team-based approach to preventive care services. However, only PARENT has randomized controlled trial (RCT) evidence of improvements in both the receipt of preventive care services and decreased emergency department (ED) use.

The central element of PARENT is the Parent Coach, a health educator who meets one-on-one with the family for approximately 15-20 minutes, depending on the needs of the parent, at the time of the WCC visit. The Parent Coach receives 4-6 weeks of WCC Parent Coach training and provides (1) anticipatory guidance (counseling and education on a broad variety of parenting-related topics), (2) psychosocial screening and community resource referral, and (3) developmental and behavioral surveillance, screening, and guidance. Parents complete a previsit screening questionnaire to help the Parent Coach prioritize their time with the family. After the family meets with the Parent Coach, the pediatrician enters the examination room to conduct the physical examination, address any clinical concerns, and provide additional guidance to parents regarding any concerns identified by the Parent Coach. Parents also receive weekly automated text messages that reinforce key health-related, age-specific guidance and education; these text messages are also designed to promote parental engagement in WCC guidance between visits.

In an initial pilot RCT of PARENT among 251 low-income families in 2 urban areas, we found strong and consistent intervention effects on the quality of preventive care provided to families and on reducing ED use (Table 1) [18,19].

**Table 1:** Parent-focused Redesign for Encounters, Newborns to Toddlers intervention pilot trial findings (n=226).

RCT^a^ results			Control		Intervention		Effect size (Cohen *d*)		*P* value
**Use, n (%)**									
	Well-visits up-to-date	84 (75.7)		86 (74.8)		N/A^b^		.88	
	Two or more sick visits	44 (39.6)		43 (37.7)		N/A		.77	
	Two or more emergency department visits	24 (21.6)		12 (10.4)		0.47		.02	
**Receipt of WCC^c^** **services, mean (SD)**									
	Anticipatory guidance	77.4 (24.5)		89.3 (12.9)		0.49		<.001	
	Health information	89.6 (22.2)		96.3 (13.8)		0.30		.008	
	Social needs assessment	77.9 (29.0)		94.9 (13.5)		0.59		<.001	
**Receipt of WCC services, n (%)**									
	Developmental screening	90 (81.1)		106 (92.2)		0.12		.01	
	Parent concerns addressed	59 (73.8)		83 (90.2)		0.28		.005	
**Experiences of care, mean (SD)**									
	Family-centered care	92.4 (13)		96.3 (8.2)		0.30		.008	
	Helpfulness of care	82.1 (19.4)		91.3 (12.3)		0.47		<.001	
	Overall rating of care	91.7 (11.6)		94.5 (9.8)		0.24		.049	

^a^RCT: randomized controlled trial.

^b^N/A: not applicable.

^c^WCC: well-child care.

### Objectives

To examine the effectiveness of PARENT as a potential evidence-based, financially sustainable model for WCC delivery, we are conducting a large, cluster RCT, in partnership with 10 clinics that are part of 2 large, multisite FQHCs. This study has 2 major phases. In phase 1, we used a community engagement and intervention implementation process [17,21-23] to guide the intervention adaptation process, Parent Coach training, practice workflow, and intervention implementation in the practices. In phase 2 (described in this protocol), we are conducting a cluster RCT of PARENT (practice-level randomization) to determine the effects on WCC quality, use, and clinician efficiency, and its costs and cost-offsets.

The cluster RCT has the following study aims and hypotheses:

- Aim 1: measure the effect of PARENT on the receipt of nationally recommended WCC services and parent experiences of care.
- Hypothesis 1: PARENT will improve the receipt of WCC services and parent experiences of care.
- Aim 2: determine the effects of PARENT on WCC, urgent care, ED use, and net costs.
- Hypothesis 2a: PARENT will result in improved up-to-date rates for WCC visits and reduced ED use.
- Hypothesis 2b: in a cost analysis, we demonstrate that the direct intervention costs of PARENT are offset by net reductions in ED use.
- Aim 3: examine the effect of PARENT on clinician time allocation for WCC and urgent care visits.
- Hypothesis 3: clinicians will shift the time from providing routine WCC services in well-visits to chronic disease management and urgent care.

## Methods

### Study Design

Our community clinical partners are 2 FQHCs located in and around Tacoma, Washington and Los Angeles, California, with 4 and 6 participating clinics, respectively. Using computer-generated random allocation, the study biostatistician block-randomized the clinics to intervention (n=5) and control (n=5), stratified by state to ensure equal numbers per state across the study arm. The 5 intervention clinics are implementing PARENT for all WCC visits through the age of 2 years at their clinical site, and the control clinics are continuing usual care (clinician-directed WCC visits). A total of 940 families (94 per clinic) with infants aged ≤12 months are being enrolled and will remain in the study for a period of 12 months. Parents will complete a survey at baseline and at 6 and 12 months after enrollment. We will examine parent-reported quality of care (receipt of nationally recommended WCC services, family-centeredness of care, and parental experiences of care) and health care use (WCC, urgent care, ED, and hospitalizations), conduct a cost analysis, and conduct a time-motion study to assess clinician time allocation for efficiency. Data on exploratory measures of parent-and parenting-focused outcomes will also be collected.

### Intervention Components

The intervention components are as follows:

- Parent Coach
  The Parent Coach is a Spanish and English bilingual, bachelor’s degree level health educator hired by the FQHC, who has participated in a 6-week training led by our academic research team. The coaches hired by the clinics are bicultural, bilingual Latinx individuals who have previously worked as care coordinators or medical interpreters; they all have experience in child health fields and in working with low-income families. The training consisted of self-directed learning based on Bright Futures Guidelines, Fourth Edition [1], relationship-building with community organizations (for resource referral) near each clinical site, simulated WCC visits with trainer feedback, coach-observed WCC visits, and pediatric provider-observed WCC visits with feedback at the clinics. The Parent Coach is available at the intervention clinics to serve as the primary provider of anticipatory guidance, psychosocial screening and referral, and developmental and behavioral guidance and screening during each WCC visit.
  The Parent Coach is available at other times to answer parents’ preventive care–related questions and conduct parent follow-up calls and visit reminders during clinic business hours.
- WCC visit process
  Upon arrival, the child is registered, weighed, and measured by the medical assistant and then taken to an examination room per the usual care process. The coach then enters the patient room and uses the completed, adapted version of the Bright Futures Previsit Questionnaire (completed in the waiting room or examination room) to guide the WCC visit [24]. The coach discusses the parents’ priorities for the visit, addressing parental concerns on topics such as feeding, sleeping, parenting, safety, and other issues. Next, the coach reviews any red flags from the questionnaire, conducts social needs and psychosocial screening, and provides any needed community resource referrals. Finally, the coach reviews developmental milestones or the structured developmental screen, if used at that visit, and addresses any behavioral concerns that the parent has.
  The Parent Coach documents the visit by the family in the electronic health record, highlighting the results of developmental and behavioral screening and any issues that the clinician needs to review at the top of the encounter page. The Parent Coach spends 15-20 minutes with the parent based on parent needs.
  After the Parent Coach completes their time with the family, the clinician enters the patient room, conducts a physical examination, and reviews the Parent Coach’s notes within the electronic health record. The clinician addresses any Parent Coach’s findings that need further clinical investigation (eg, concern for speech delay on developmental surveillance), any additional parent concerns, and any chronic or urgent care issues. In cases where the Parent Coach identifies concerns that need immediate attention from the clinician, the Parent Coach will directly communicate with the clinician about the parents’ needs and follow-up plan (warm hand-off).
- Healthy text messages
  Parents enrolled in the intervention clinics are offered a child health-focused text messaging service at study enrollment. Research staff help parents enroll into the text messaging service by texting their cell phone number, language preference (English or Spanish), clinic location, and child’s date of birth to a number provided by Healthy-TXT, a text messaging service (Healthy-TXT LLC). The library of text messages was adapted from Healthy-TXT to meet the needs of the FQHCs. If the parent chooses to sign up for the text message service (parents can be enrolled into the study and decline text messages), they receive weekly messages focused on age-appropriate anticipatory guidance, health education, and reminders for WCC visits. These messages are tailored to the child’s age and parent’s language preference (Spanish or English). Most messages include a link to an educational website (eg, healthychildren.org) with a video or written information on that specific topic. Some messages include the clinic’s telephone number for visit scheduling, the Parent Coach’s number, or other information (eg, poison control hotline). At any time, parents can text “STOP” to end the service.

### Procedures

#### Patient Recruitment and Enrollment at Intervention and Control Clinics

Parents or legal guardians checking in for a WCC visit or follow-up visit for an infant aged ≤12 months are approached by research staff in the waiting room. The research staff obtain a schedule of WCC visits via an encrypted email sent by the clinic staff. The schedule includes the date and time of all WCC visits for infants aged ≤12 months—no protected health information is included in the schedule. The research staff determine which clinics to visit for recruitment based on this schedule, using a systematic approach. On the day of clinic recruitment, research staff, clinic staff, and front desk staff closely coordinate recruitment. The front desk staff verifies the WCC visits each day with the research staff and obtains verbal permission from the parent (or legal guardians) to be approached by research staff at the clinic that day to talk about the study. Those who agree are approached while waiting for their WCC visit.

#### Participants and Enrollment Procedures

For families who are approached in clinics, the study staff explain the study and screen for the following eligibility criteria: (1) be an adult (aged ≥18 years) parent or legal guardian of a child aged ≤12 months arriving for a visit, (2) have no plans to change clinic providers for the next 12 months, and (3) have English or Spanish language proficiency. Eligible families provide informed consent and parental permission. For multiple gestations, one of the infants is selected at random as the index child enrolled in the study. Children with special health care needs are not excluded from the study; these children generally receive the same recommended preventive care services.

Contact information is collected from the enrolled parent or legal guardian and spouse or partner (home and cell phone and email) to contact them to complete the baseline survey, if needed. The information is verified at each subsequent research contact for the follow-up surveys at 6 and 12 months from the date of enrollment. We also ask parents to provide the contact information of 3 relatives or friends who will always know their whereabouts.

Although this study involves direct interaction with economically and educationally disadvantaged persons, we are careful to minimize the risk of coercion or undue influence. The recruitment process for the study is completely voluntary, and families are informed that they can withdraw from the study at any point. Those who agree to participate receive a cash or gift card incentive for each survey. Consent forms have been so written as to be understood by those with limited reading or writing proficiency in both English and Spanish. However, if a participant is unable to read the consent form, the research assistant reads the form aloud, and a witness initials the form, acknowledging that the terms of agreement have been read to the participant.

### Data Collection

We shall conduct 3 waves of parent survey data collection (baseline, 6-month and 12-month), and will review the child’s electronic chart at 12 months follow-up. Interviewers are not blinded to the group assignment.

- Baseline survey (at enrollment, in person or via phone): Upon enrollment, parents participate in a 35-minute survey for the collection of baseline demographic data on the infant, parent, household, child’s medical history, and health care use during the 3 months before the survey.
- Six-month survey: Research staff conduct a 15-minute phone survey that updates the child’s medical history, household changes, and health care use since enrollment (eg, ED use). The 6-month survey is designed to increase participant retention over the 12-month study period, by providing an opportunity for engagement at 6 months, and is designed to improve data accuracy by using a 6-month recall period, rather than a 12-month recall, for use-related questions.
- Twelve-month survey: All participants will be asked to complete a 40-minute survey by phone. This survey administration includes questions from the Promoting Healthy Development Survey, a parent survey that assesses the receipt of nationally recommended WCC services [25]. It is endorsed by the National Quality Forum and has been used by 10 State Medicaid agencies, 4 health plans, 38 pediatric practices, and nationally through the National Survey of Early Childhood Health to collect data for over 45,000 children [26]. It is available in English and Spanish and is written at an 8th grade reading level. It has strong construct validity (mean factor loading: 0.69) and internal consistency (mean Cronbach α=.80) [27]. A previous study using this survey revealed that quality measure scores for children in 3 health plans ranged from 17 to 75 (on a 100-point scale) and varied significantly across health plans [27].

We will use the Promoting Healthy Development Survey to measure the parent-perceived receipt of recommended WCC services. Questions are also included on social determinant screening and referral drawn from the Promoting Healthy Development Survey and health care use since the 6-month survey. We will also use items on overall satisfaction with care from the Consumer Assessment of Health care Providers and Systems Health Plan Survey [28,29] and family-centeredness of care from the National Survey of Children’s Health [30]. We shall collect data on exploratory measures of parental mental health [31] and parenting behaviors [25,30].

For the 6- and 12-month follow-up surveys, we will contact the study participants 2 weeks before the due date. If we are unable to reach them by the due date, we will continue calling them up to 3 months after the due date to complete the survey. After completing the 3 waves of survey data collection, we will conduct chart reviews to extract the number of clinic visits (total, WCC, and acute care) and immunization records. The parent or legal guardian will receive a gift card or cash incentive after completing the baseline survey (US $30) and follow-up surveys at 6 months (US $20) and 12 months (US $40) after enrollment.

Finally, to assess changes in clinician time and WCC content covered during visits, we will conduct a time-motion study, consisting of direct observations of clinic teams (clinician and Parent Coach) at 20 randomly selected WCC visits at each clinical site (intervention and control) before and during intervention implementation. This data collection is not part of the RCT but is a supplemental observational study that will allow us to understand how the visit differs with and without the Parent Coach. A total of 200 time-motion study visits will be observed during the first year for the control and intervention clinics. During the fourth and fifth project years, we will observe an additional 200 time-motion study visits for intervention and control clinics.

### Safeguarding

To ensure quality control of data, the study team member who collects the data enters the data into the protected database, and another team member validates the data entry. In addition, to ensure confidentiality, parent or child participant data are entered directly into a REDCap (Research Electronic Data Capture; Vanderbilt University) database on password-protected laptops and electronic tablets. Any data initially collected from a hard copy is transferred to the secure database. Hard copy documents are kept in locked cabinets in a locked office. Identifiable information, such as consent forms, is kept separately from data collection forms to ensure deidentification of the data. Hard copy documents collected from clinics and transported to study offices are kept in secure document holders to protect against breaches of confidentiality.

### Main Outcomes

#### Overview

Our dual primary outcome measures are *the receipt of preventive care services* for anticipatory guidance received and ED use. Secondary outcomes include other measures for the receipt of preventive care services, use, experiences of care, costs, and allocation of provider time. Multimedia Appendix 1 [1-41] presents a table of all study measures.

#### Primary Outcomes Selection

Our primary outcomes are receipt of anticipatory guidance and ED use. We will select a WCC quality measure and a health care use measure to represent the outcomes most important to the stakeholders within WCC (parents, providers, and payers). We considered that costs may be most important for the sustainability of PARENT, but the receipt of WCC services may be most important to its acceptability with parents and pediatric providers. We do not consider downstream child or parent health outcomes as primary measures, but we will conduct an analysis of exploratory measures such as effective parenting and parent mental health that have been related to child outcomes [32-36].

### Analysis

#### Overview

Once all data are collected, we will compile descriptive statistics on all outcome variables, composite scores, and covariates. We will report means and SDs (or medians and IQRs as appropriate) for continuous variables, create graphical displays to visualize distributions, and transform variables with nonnormal distributions, such as cost offsets. Next, we will use multivariable analyses to examine the differences between the control and intervention groups at 12 months on the measures described above. From these analyses, we will estimate the intervention effects. An intent-to-treat approach rather than a per-protocol approach will be used; that is, parents will be analyzed according to the assigned group regardless of deviations from the study protocol. Missing outcome data will be handled using a strategy described by White et al [37]. Observations with missing values will be excluded from the initial analysis (complete case analysis) [37]. We will examine and report the patterns and plausible causes of any missing data. This will then inform additional analyses using multiple imputation, comparing the effects of different plausible assumptions about the nature of the missing data. Results with and without multiple imputations will be presented. Additional sensitivity analyses will be conducted by performing the analyses with and without outliers and influential data points. All tests will be 2-sided, and *P* values <.05, will be considered statistically significant.

#### Primary Outcome 1: Anticipatory Guidance

We will analyze anticipatory guidance based on data collected at 12 months after enrollment. We will assess the intervention effect on anticipatory guidance via linear mixed-effects models that include random effects to account for clustering by clinic. The outcome may be transformed before fitting the model if nonnormality is observed. The analysis will include intervention status as the main independent variable and may be adjusted for potential confounding factors that differ between the 2 groups at baseline. We will also test for possible interaction effects between the intervention and these covariates. All other outcome variables to measure experiences of care and receipt of other nationally recommended WCC services are regarded as secondary. Each outcome will be analyzed using linear or generalized linear mixed effects models.

#### Primary Outcome 2: Health Care Use

Intervention effects on health care use (ED visits, hospitalizations, and urgent care visits) will be assessed using generalized linear mixed-effects models for binary or count data. The model will include random effects to capture data clustering within the clinic; the primary independent variable will be intervention status. The analysis will be adjusted for any baseline child, parent, or household characteristics that differ between the control and intervention groups. We will also test for interaction between child age and the intervention, as well as between the intervention and any covariates that differ across study arms at baseline.

### Cost Analysis

We will conduct our analysis from the perspective of the health sector and consider intervention costs (direct and indirect) and cost offsets [38].

#### Intervention Costs

We will distinguish between start-up costs (eg, Parent Coach training), fixed annual maintenance costs (eg, text message service), and the marginal cost of adding patients to the existing intervention (eg, additional time spent by Parent Coaches if WCC caseloads increase). The primary source of direct intervention costs is staff time, particularly the Parent Coach time. Staff time will be tracked during randomly selected weeks throughout the study using intervention service logs valued at total compensation (salary+benefits). Other intervention costs, such as the opportunity costs of using clinic space (included at the value of its opportunity cost) and parents’ time (valued using national average wage rates from the Bureau of Labor Statistics) will be tracked by study accounting procedures. We will exclude evaluation-related costs from our estimates.

#### Cost Offsets

To explore health care use, we will collect parent survey data on the use of services that we hypothesize may be affected by PARENT. This includes additional services (eg, more referrals for behavioral problems because of better identification) and cost offsets (eg, reduced ED visits). We will construct parent-specific measures as the weighted sum of the number of units used in each service category, weighted by the unit cost of that type of service. We will use national estimates of unit costs by condition and age group, such as the Disease Expenditure Study estimates of unit cost per outpatient visit, ED visit, and hospital stay [39]. To test whether the intervention leads to budget neutrality or even net cost savings, we will estimate the mean difference in nonintervention costs between intervention and control using multivariate regression methods. The analysis may be conducted with the log of cost or other transformation, as the distribution of costs often has a long right tail representing a few patients with high expenditures. We will then compare the average intervention cost per child with the estimated savings in nonintervention costs. Although the average intervention cost could increase if the clinic is not large enough to use Parent Coaches at their optimal capacity, we will test the sensitivity of our conclusions to varying assumptions about the size of the patient panel versus the minimum full-time equivalent level at which the Parent Coach can be hired.

#### Time-Motion

We will collect time-motion data on at least 20 randomly selected well-visits per clinic, before and after PARENT implementation. In-person visit observations will provide the number of minutes (total and per visit) of clinician time and Parent Coach time in rooms with family for each WCC visit from 2-24 months, and data on visit content (discussion of preventive, chronic, or urgent care issues) of clinician WCC time with families (enrolled participants for WCC visits). We will examine clinician and Parent Coach time allocation for WCC visits from ages 0 to 2 years, comparing the mean time for each activity before and after the intervention implementation using linear mixed-effects models. This model will include random effects for the clinic to account for clustering, a binary variable as a primary predictor to indicate whether measurement occurred before or after intervention implementation, and an indicator for the group (intervention vs control). The analysis will also be adjusted for other factors as deemed appropriate. We will qualitatively describe the intervention versus control differences in the content discussed during visits.

### Power Assessment

The power calculation is based on WCC quality (anticipatory guidance) and ED visits (2 or more), with a 1:1 randomization of 10 sites to the intervention and control groups. We will use the mean and SD of anticipatory guidance and the rates of 2 or more ED visits, as observed in our pilot RCT intervention and control groups. For anticipatory guidance, we will use the larger SD from the 2 groups to obtain a conservative power estimation for this variable. The calculation assumes an intraclass correlation of 0.01 based on previous cluster RCTs of similar delivery systems among similar populations, and the fact that the data analysis may be adjusted for important baseline covariates when comparing the intervention with control groups [40,41]. A conservative 20% dropout rate is assumed at the patient level; in other words, the retention of at least 75 of the 94 participants enrolled per site. Two-sided tests will be used with a type I error rate of 0.05. With a total of 94 participants enrolled per site, we will have at least 80% power to detect the anticipated intervention effect for both anticipatory guidance and ED visits, assuming a 20% dropout rate at the patient level.

For the time-motion study analysis, time measurements for 20 randomly selected participant visits (at each clinic, before and after implementation) will provide 80% power to detect an effect size of 0.30, using a 2-sided test at a significance level of .05, assuming an intraclass correlation of 0.01, and interperiod correlation of 0.01. This is based on an average provider time of 15 minutes (SD 5 minutes) in the control group, and a detectable intervention-related change of approximately 2 minutes 2 seconds.

## Results

Participant recruitment began in March 2019. After recruitment, 6- and 12-month follow-up surveys will be completed. As of August 30, 2021, we have enrolled a total of 916 participants.

## Discussion

### Principal Findings

PARENT is an innovative WCC delivery model designed to meet the needs of low-income families. Promising preliminary findings suggest that PARENT may be a more effective system for the delivery of WCC, providing family-centered, comprehensive preventive care for infants and toddlers in low-income communities. This study contributes to the assessment of the effectiveness of PARENT across multiple practices, with a larger population of families and multiple Parent Coaches.

### Conclusions

Through this research, we will examine PARENT across a larger number of practices, assess its effects on receipt of nationally recommended WCC services, parent experiences of care, health care use, costs, and impact on clinicians’ allocation of time, and explore its effect on parent outcomes known to be associated with subsequent child outcomes.

If PARENT is shown to improve quality, improve experiences of care, and prove financially viable, it can be scaled to FQHCs and other practices nationally.

## Appendix

Multimedia Appendix 1 Study measures.

## Abbreviations

ED: emergency department
FQHC: federally qualified health center
PARENT: Parent-focused Redesign for Encounters, Newborns to Toddlers
RCT: randomized controlled trial
REDCap: Research Electronic Data Capture
WCC: well-child care
