# Supplementary material for: A Parent Coach–Led Model of Well-Child Care for Young Children in Low-Income Communities: Protocol for a Cluster Randomized Controlled Trial
Source: JMIR Res Protoc. 2021 Nov 25;10(11):e27054. doi: 10.2196/27054 (PMC8663704; doi:10.2196/27054)
Supplement: Multimedia Appendix 1 [file resprot_v10i11e27054_app1.docx]

1. Hagan JF, Shaw JS, Duncan PM, editors. Bright Futures: Guidelines for Health Supervision of Infants, Children and Adolescents. 4th ed. Elk Grove, IL: American Academy of Pediatrics; 2017.

2. Bethell C, Reuland C, Schor E, Abrahms M, Halfon N. Rates of parent-centered developmental screening: disparities and links to services access. Pediatrics. 2011;128(1):146-55.

3. Chung PJ, Lee TC, Morrison JL, Schuster MA. Preventive care for children in the United States: Quality and barriers. Ann Rev Public Health. 2006;27:491-515.

4. Schuster M, Duan N, Regalado M, Klein D. Anticipatory guidance: What information do parents receive? What information do they want? Arch Pediatr Adol Med. 2000;154:1191-8.

5. Bethell C, Reuland CH, Halfon N, Schor EL. Measuring the quality of preventive and developmental services for young children: National estimates and patterns of clinicians' performance. Pediatrics. 2004;113(6 Suppl):1973-83.

6. Halfon N, Regalado M, Sareen H, Inkelas M, Reuland CH, Glascoe FP, et al. Assessing development in the pediatric office. Pediatrics. 2004;113(6 Suppl):1926-33.

7. Norlin C, Crawford MA, Bell CT, Sheng X, Stein MT. Delivery of well-child care: a look inside the door. Acad Pediatr. 2011;11(1):18-26.

8. Freeman BK, Coker TR. Six Questions for Well-Child Care Redesign. Academic Pediatrics. 2018;18(6):609-19.

9. Olson LM, Inkelas M, Halfon N, Schuster M, O'Connor KG. Overview of the content of health supervision for young children: Reports from parents and pediatricians. Pediatrics. 2004;113(6 Suppl):1907-16.

10. Coker TR, Thomas T, Chung PJ. Does well-child care have a future in pediatrics? Pediatrics. 2013;131 Suppl 2(S1):S149-59.

11. Coker TR, Windon A, Moreno C, Schuster MA, Chung PJ. Well-child care clinical practice redesign for young children: a systematic review of strategies and tools. Pediatrics. 2013;131 Suppl 1(Supplement 1):S5-25.

12. Coker T, Casalino LP, Alexander GC, Lantos J. Should our well-child care system be redesigned? A national survey of pediatricians. Pediatrics. 2006;118(5):1852.

13. Mooney K, Moreno C, Chung PJ, Elijah J, Coker TR. Well-child care clinical practice redesign at a community health center: provider and staff perspectives. J Prim Care Community Health. 2014;5(1):19-23.

14. Workgroup on Bright Futures Periodicity Schedule and Committee on Practice and Ambulatory Medicine. 2017 recommendations for preventive pediatric health care. Pediatrics. 2017:e20170254.

15. Coker TR, Chung PJ, Cowgill BO, Chen L, Rodriguez MA. Low-income parents' views on the redesign of well-child care. Pediatrics. 2009;124(1):194-204.

16. Radecki L, Olson LM, Frintner MP, Tanner JL, Stein MT. What do families want from well-child care? Including parents in the rethinking discussion. Pediatrics. 2009;124(3):858-65.

17. Coker TR, Moreno C, Shekelle PG, Schuster MA, Chung PJ. Well-child care clinical practice redesign for serving low-income children. Pediatrics. 2014;134(1):e229-39.

18. Coker TR, Chacon S, Elliott MN, Bruno Y, Chavis T, Biely C, et al. A parent coach model for well-child care among low-income children: a randomized controlled trial. Pediatrics. 2016:peds. 2015-3013.

19. Mimila NA, Chung PJ, Elliott MN, Bethell CD, Chacon S, Biely C, et al. Well-Child Care Redesign: A Mixed Methods Analysis of Parent Experiences in the PARENT Trial. Acad Pediatr. 2017;17(7):747-54.

20. Minkovitz CS, Strobino D, Mistry KB, Scharfstein DO, Grason H, Hou W, et al. Healthy Steps for Young Children: Sustained results at 5.5 years. Pediatrics. 2007;120(3):e658-68.

21. Contreras S, Porras-Javier L, Zima BT, Soares N, Park C, Patel A, et al. Development of a Telehealth-Coordinated Intervention to Improve Access to Community-Based Mental Health Care. Ethnicity and Disease. 2018;28(Suppl 2):457-66.

22. Jones KA, Do S, Porras-Javier L, Contreras S, Chung PJ, Coker TR. Feasibility and Acceptability in a Community-Partnered Implementation of CenteringParenting for Group Well-Child Care. Academic pediatrics. 2018;18(6):642-9.

23. Patel M, Andrea N, Jay B, Coker TR. A Community-Partnered, Evidence-Based Approach to Improving Cancer Care Delivery for Low-Income and Minority Patients with Cancer. Journal of community health. 2019;44(5):912-20.

24. American Academy of Pediatrics. Bright Futures Tool and Resource Kit, Second Edition [Available from: <https://brightfutures.aap.org/materials-and-tools/tool-and-resource-kit/Pages/default.aspx>.

25. Bethell C, Reuland C, Latzke B. The Promoting Healthy Development Survey (PHDS-PLUS): Implementation guidelines for Medicaid and other settings. The Commowealth Fund; 2005 November.

26. Child and Adolescent Health Measurement Initiative. Preventive Services for Young Children: Promoting Healthy Development Survey 2006 [Available from: <http://cahmi.org/pages/Sections.aspx?section=8>.

27. Bethell C, Peck C, Schor EL. Assessing health system provision of well-child care: the Promoting Healthy Development Survey. Pediatrics. 2001;107:1094-.

28. Agency for Healthcare Research and Quality. Topics and related items in the CAHPS Health Plan Survey 4.0 Core Questionnaires. Rockville, MD: AHRQ; 2006 [Available from: <https://www.cahps.ahrq.gov/content/products/PDF/PROD_HP4_Core.htm>.

29. CAHPS Clinician & Group Survey. Reporting Measures for the CAHPS Clinician & Group Survey 2010 [Available from: <https://www.communitycarenc.org/media/files/309-4_cg_reporting_measures_4pt.pdf>.

30. Bramlett M, Blumberg S, Zablotsky B, George J, Ormson A, Frasier A, et al. Design and Operation of the National Survey of Children's Health, 2011-2012. Vital and health statistics Ser 1, Programs and collection procedures. 2017(59):1-256.

31. Hays RD, Schalet BD, Spritzer KL, Cella D. Two-item PROMIS® global physical and mental health scales. Journal of patient-reported outcomes. 2017;1(1):2-.

32. Reyno SM, McGrath PJ. Predictors of parent training efficacy for child externalizing behavior problems–a meta‐analytic review. Journal of Child Psychology and Psychiatry. 2006;47(1):99-111.

33. Jones TL, Prinz RJ. Potential roles of parental self-efficacy in parent and child adjustment: A review. Clinical Psychology Review. 2005;25(3):341-63.

34. Petterson SM, Albers AB. Effects of poverty and maternal depression on early child development. Child Development. 2001:1794-813.

35. Goodman SH, Rouse MH, Connell AM, Broth MR, Hall CM, Heyward D. Maternal depression and child psychopathology: a meta-analytic review. Clin Child Fam Psychol Rev. 2011;14(1):1-27.

36. Brennan PA, Hammen C, Andersen MJ, Bor W, Najman JM, Williams GM. Chronicity, severity, and timing of maternal depressive symptoms: relationships with child outcomes at age 5. Developmental psychology. 2000;36(6):759.

37. White IR, Horton NJ, Carpenter J, Pocock SJ. Strategy for intention to treat analysis in randomised trials with missing outcome data. Bmj. 2011;342.

38. Neumann P, Ganiats T, Russell L, Sanders G, Siegel J, editors. Cost-Effectiveness in Health and Medicine. Second ed. Oxford, New York: Oxford University Press; 2016.

39. Dieleman JL, Cao J, Chapin A, Chen C, Li Z, Liu A, et al. US Health Care Spending by Payer and Health Condition, 1996-2016. JAMA. 2020;323(9):863-84.

40. Garg A, Toy S, Tripodis Y, Silverstein M, Freeman E. Addressing social determinants of health at well child care visits: a cluster RCT. Pediatrics. 2015;135(2):e296-304.

41. Garbutt JM, Highstein G, Yan Y, Strunk RC. Partner randomized controlled trial: study protocol and coaching intervention. BMC Pediatr. 2012;12(1):42.

**Appendix. Study Measures**

| **Outcome Measures** | **Source** | **Survey Items*** |
| --- | --- | --- |
| **1. Utilization Measures** | | |
| a. WCC up-to-date (UTD) | Chart review | N/A |
| b. Sick/urgent visits | Chart review | N/A |
| c. Immunizations UTD | Chart Review | N/A |
| d. Emergency department (ED) | PHDS | 1 |
| e. Hospitalizations | PHDS | 1 |
| f. Other Utilization (e.g., early intervention referrals, maternal mental health, breastfeeding consultation) | Utilization survey | 5 |
| **2. Receipt of Preventive Care Service** | | |
| a. Anticipatory guidance (AG) | PHDS | 15-18 |
| b. Psychosocial assessment | PHDS | 6 |
| c. Developmental screening | PHDS | 3 |
| d. Developmental concerns addressed | PHDS | 2 |
| e. Health Information | PHDS | 3 |
| **3. Experiences of Care** | | |
| a. Helpfulness of care | PHDS | 4 |
| b. Family centeredness of care | NSCH | 5 |
| c. Overall rating of care | CAHPS | 1 |
| **4. Costs** | | |
| a. Direct and indirect intervention costs | Study logs, Accounting | N/A |
| b. Utilization | Parent reported health care use, WCC clinic chart review | N/A |
| c. Unit cost | National estimates such as disease expenditure study | N/A |
| **5. Time-Motion Study (TMS)** |  |  |
| a. Total visit time | TMS | N/A |
| b. Time with clinician | TMS | N/A |
| c. Time with coach | TMS | N/A |
| d. Time spent discussing WCC topics (anticipatory guidance, social needs, and development/behavior) | TMS |  |
| **6. Parent Measure** |  |  |
| Parenting positive behaviors | NSCH | 2 |
| Parental Physical and Mental Health | PROMIS | 4 |
| * # of survey items  PHDS: Promoting Healthy Development Survey; NSCH: National Survey of Children’s Health; CAHPS: Consumer Assessment of Healthcare Providers and Systems (CAHPS^®^); TMS: Time Motion Study; PROMIS: Patient-Reported Outcomes Measurement Information System | | |
